# Quantum Chemical Investigation on the Material Properties of Al-Based Hydrides XAl_2_H_2_ (X = Ca, Sr, Sc, and Y) for Hydrogen Storage Applications

**DOI:** 10.3390/ma18153521

**Published:** 2025-07-27

**Authors:** Yong Guo, Rui Guo, Lei Wan, Youyu Zhang

**Affiliations:** 1 Department of Physics, Shanxi Agricultural University, Jinzhong 030801, China; sxauguor@163.com; 2 School of Materials Science and Engineering, Lanzhou University of Technology, Lanzhou 730050, China; leiwan@lut.edu.cn; 3 Institute of Computational (Digital) Materials, Lanzhou University of Technology, Lanzhou 730050, China; 4 Jiuquan Iron and Steel (Group) Corporation, Jiuquan 735000, China; zhangyouyu@jiugang.com

**Keywords:** aluminum hydrides, hydrogen storage, mechanical properties, phonon dispersion, thermodynamic properties

## Abstract

Aluminum–hydrogen compounds have drawn considerable interest for applications in solid-state hydrogen storage. The structural, hydrogen storage, electronic, mechanical, phonon, and thermodynamic properties of XAl_2_H_2_ (X = Ca, Sr, Sc, Y) hydrides are investigated using density functional theory. These hydrides exhibit negative formation energies in the hexagonal phase, indicating their thermodynamic stability. The gravimetric hydrogen storage capacities of CaAl_2_H_2_, SrAl_2_H_2_, ScAl_2_H_2_, and YAl_2_H_2_ are calculated to be 1.41 wt%, 0.94 wt%, 1.34 wt%, and 0.93 wt%, respectively. Analysis of the electronic density of states reveals metallic characteristics. Furthermore, the calculated elastic constants satisfy the Born stability criteria, confirming their mechanical stability. Additionally, through phonon spectra analysis, dynamical stability is verified for CaAl_2_H_2_ and SrAl_2_H_2_ but not for ScAl_2_H_2_ and YAl_2_H_2_. Finally, we present temperature-dependent thermodynamic properties. This research reveals that XAl_2_H_2_ (X = Ca, Sr, Sc, Y) materials represent promising candidates for solid-state hydrogen storage, providing a theoretical foundation for further studies on XAl_2_H_2_ systems.

## 1. Introduction

The exploitation of traditional energy resources, such as coal, natural gas, and oil, has propelled societal progress. However, this dependence has led to numerous challenges, including environmental deterioration, the depletion of energy reserves, rising carbon emissions, and the worsening of climate change [1,2]. To address these issues, increasing attention is being directed toward the exploration of abundant and clean alternative energy sources [3]. Hydrogen energy, characterized by its high calorific value, non-polluting properties, and abundant availability, has emerged as one of the most promising alternatives [4]. Hydrogen storage, as one of the four key components of the hydrogen energy economy (the others being hydrogen production, transportation, and utilization), is the primary factor constraining the widespread adoption of hydrogen energy [5]. Despite the presence of well-established hydrogen storage techniques such as compression and liquefaction, solid-state hydrogen storage has emerged as a highly promising field, drawing increasing research interest owing to its potential to enhance both safety and storage density [6,7]. In this context, solid-state hydrogen storage materials have garnered significant interest as promising sustainable energy solutions in recent years [8].

Among the wide array of these materials, the light metal hydrides, particularly those represented by aluminum-based hydrogen compounds [9,10,11,12,13,14,15,16], have consistently received considerable focus and sustained research attention owing to their high hydrogen capacity, reversible storage properties, and lower cost. In 2000, Gingl et al. [17] first reported the synthesis of a novel aluminum–hydrogen compound, SrAl_2_H_2_, which marked the discovery of the first Zintl phase hydride. As a critical class of solid-state materials, Zintl phases provide a theoretical framework for designing multifunctional compounds with diverse physical properties [18]. For instance, these materials exhibit remarkable characteristics such as thermoelectricity, superconductivity, and anomalous/spin Hall effects, rendering them highly attractive for applications in emerging fields like spintronics and optoelectronics [19]. The SrAl_2_H_2_ compound crystallizes in a hexagonal crystal structure containing a two-dimensional polyanionic [Al_2_H_2_]^2−^ layer, where one aluminum atom is covalently bonded to each hydrogen atom. Björling et al. systematically investigated the hydrogenation reaction of the intermetallic compound AeE_2_ (Ae = Ca, Sr, Ba; E = Al, Ga, In), synthesizing and characterizing SrAl_2_H_2_, in addition to two isomorphic hydrides SrGa_2_H_2_ and BaGa_2_H_2_ [20]. Additionally, the first-principles method was employed to calculate the energy of these compounds, thereby theoretically confirming their stability. Lee et al. performed a comprehensive investigation into the lattice vibrational properties of SrAl_2_H_2_ and SrAlSiH by combining experimental and theoretical methods, revealing that the stability of solid aluminum hydride is inversely related to the strength of Al-H bonding [21]. Based on the results of the electronic structure and phonon spectral frequency calculated from first principles, Subedi et al. evaluated the chemical bonding properties of compounds such as SrAl_2_H_2_, SrGa_2_H_2_, and BaGa_2_H_2_, and they found that these compounds display a combination of covalent and ionic bonding characteristics [22].

To the best of our knowledge, apart from one report on the BaAl_2−x_Si_x_H_2−x_ (0.4 < x < 1.6) series hydrides [23], which are intermediate in composition and structure between BaSi_2_ and BaAl_2_H_2_, very few additional aluminum–hydrogen compounds with the same type of structure have been documented, either experimentally or computationally, since the synthesis of SrAl_2_H_2_ was reported in 2000. Moreover, there is a notable scarcity of research specifically focusing on their hydrogen storage capacity. On the one hand, investigation into the novel structure of aluminum hydride compounds facilitates the expansion of hydrogen storage types and paves the way for practical material innovations. On the other hand, analyzing complex hydrides, such as SrAl_2_H_2_, provides deeper insights into the atomic bonding mechanisms within these materials [24].

To the best of our knowledge, among the XAl_2_H_2_ family, only SrAl_2_H_2_ has been experimentally synthesized to date, with subsequent theoretical studies primarily focusing on Al-H bonding interactions. However, for the isomorphic XAl_2_H_2_-type compounds, no prior investigations have systematically examined their physical properties, particularly in the context of hydrogen storage applications. Against this background, we selected Ca, Sc, and Y as candidate elements for our study. These elements are located near Sr in the periodic table (either in the same period or adjacent groups) and exhibit similar electronic configurations and chemical bonding characteristics, indicating their potential to form stable compounds analogous to SrAl_2_H_2_. Guided by this rationale, we investigate the Al-based hydrides XAl_2_H_2_ (X = Ca, Sr, Sc, Y) as potential hydrogen energy carriers. Using first-principles calculations, we conduct a systematic analysis of their structural, hydrogen storage, electronic, mechanical, lattice dynamical, and thermodynamic properties.

## 2. Computational Details

The structural and electronic properties of XAl_2_H_2_ (X = Ca, Sr, Sc, Y) hydrides were systematically investigated using first-principles density functional theory (DFT) [25] calculations implemented in the Wien2k package (version: Wien2k_23) [26]. The all-electron full-potential linearized augmented plane wave (FP-LAPW) method with the generalized gradient approximation (GGA-PBE) [27] functional was employed for exchange-correlation treatment. Muffin-tin radii of 2.0 a.u. were used for metallic atoms (Ca/Sr/Sc/Y/Al), while 1.0 a.u. was applied for hydrogen atom. A cutoff energy of −6.0 Ry was set to separate valence-core states, and R_MT_·K_MAX_ = 4.0 ensured plane-wave convergence. Structural optimizations were performed for all compounds using the 2DRoptimize module within Wien2k, which is specifically designed to handle hexagonal symmetry. This module facilitates anisotropic relaxation of lattice parameters (*a* and *c*) while maintaining hexagonal symmetry constraints, enabling efficient optimization of both the unit cell volume and the *c*/*a* ratio. Internal atomic coordinates were relaxed using the MINI module within Wien2k, which employs the “reverse-communication trust-region quasi-Newton” method [28] to minimize the total energy. Both lattice parameters and atomic positions were optimized using 8000 k-points in the full Brillouin zone, with convergence thresholds set to 10^−4^ Ry for total energy and 10^−4^ e/Å^3^ for charge density. These parameters ensure consistent accuracy between structural relaxations and subsequent property calculations. Elastic properties were derived from second derivatives of energy-based calculations using the IRelast code [29], providing polycrystalline elastic moduli, Poisson’s ratio, wave velocities, and Debye temperature. Anisotropy analysis was conducted using ELATool [30]. Phonon spectra and density of states were calculated via the finite displacement method [31] implemented in Phonopy [32] (1 × 1 × 1 supercell, 5 atoms), followed by thermodynamic property determination through harmonic approximation analysis of phonon density of states.

## 3. Results and Discussion

### 3.1. Structural and Hydrogen Storage Properties

**Figure 1:**
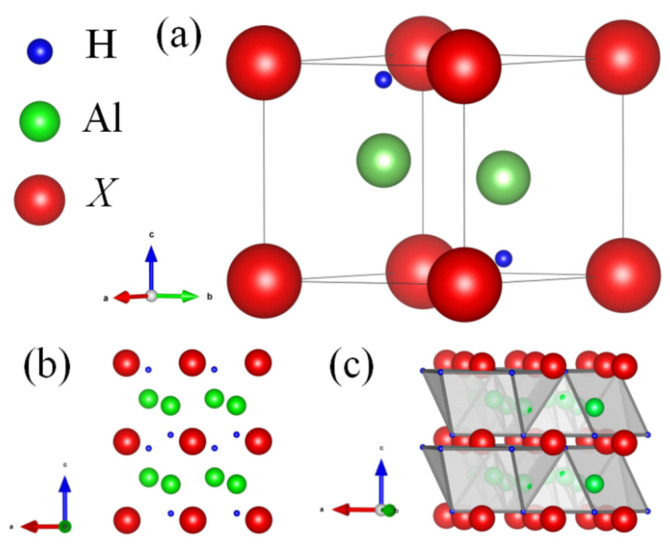
(Color online.) The crystal structure of XAl2H2 (X = Ca, Sr, Sc, Y): (**a**) the side view of 1 × 1 × 1 unit cell; (**b**) the front view of 2 × 2 × 2 supercell; (**c**) the polyhedral view of 2 × 2 × 2 supercell.

The crystal structure of XAl_2_H_2_ (X = Ca, Sr, Sc, Y) hydrides, as illustrated in Figure 1a, exhibits a hexagonal structure with the P-3m1 (No. 164) space group. The unit cell comprises five atoms: the metal atom X (X = Ca, Sr, Sc, Y) occupies the 1*a* (0, 0, 0) Wyckoff site at the hexagonal corners; Al atoms occupy the 2*d* (1/3, 2/3, *Z*_Al_) Wyckoff site; and H atoms occupy another 2*d* (1/3, 2/3, *Z*_H_) Wyckoff sites. From the combination of Figure 1b,c, it can be observed that the XAl_2_H_2_ crystal structure consists of alternately stacked layers: a tetrahedral layer of hydrogen atoms (with aluminum atoms located within this tetrahedral framework) and a planar triangular layer of X atoms.

**Figure 2:**
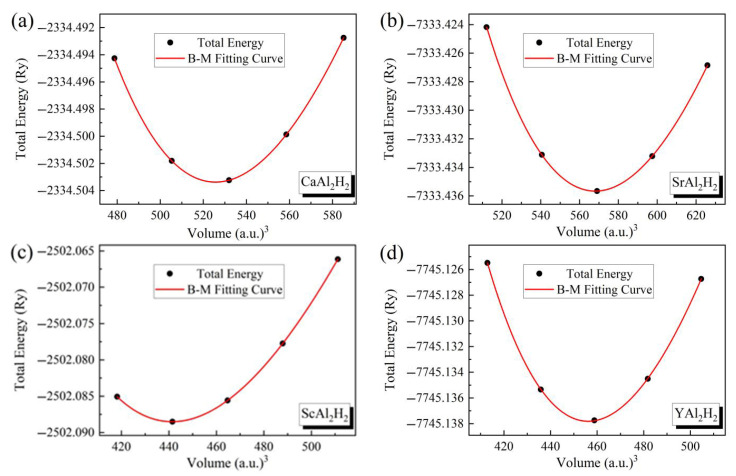
(Color online.) The total energy versus volume curve of (**a**) CaAl2H2, (**b**) SrAl2H2, (**c**) ScAl2H2 and (**d**) YAl2H2.

To determine the structural parameters of the equilibrium state for the system, the energy–volume relationship and the energy–*c*/*a* relationship have been optimized. The optimization curves are presented in Figure 2. Subsequently, the lattice constants, *c*/*a* ratio, unit cell volume *V*_0_, bulk modulus *B*_0_, and its pressure derivative *B*’ are calculated by fitting the data to the Birch–Murnaghan (B-M) equation of state, expressed as follows [33]:

(1) $$E(V)=E_{0}+\frac{9V_{0}B_{0}}{16}\left\{{\left[{\left(\frac{V_{0}}{V}\right)}^{\frac{2}{3}}-1\right]}^{3}B^{\prime }+{\left[{\left(\frac{V_{0}}{V}\right)}^{\frac{2}{3}}-1\right]}^{2}\left[6-4{\left(\frac{V_{0}}{V}\right)}^{\frac{2}{3}}\right]\right\}$$

where *E*_0_ is the total energy, *V*_0_ is the equilibrium unit cell volume, *B*_0_ is the bulk modulus, and *B*’ is the pressure derivative of the bulk modulus.

The optimized internal coordinates of Al and H atoms, as well as the shortest interatomic distances between Al and H in XAl_2_H_2_ (X = Ca, Sr, Sc, Y), are summarized in Table 1, together with the experimental [17] and theoretical [22] values for SrAl_2_H_2_. First, it is evident that for SrAl_2_H_2_, the calculated coordinates of Al and H atoms align very closely with the experimental values. The distances between nearest neighbor Al and H atoms are marginally smaller than the theoretical values reported in reference [22] but remain closer to the experimental values. This not only validates the reliability of our computational methodology but also implies that the calculated values for other compounds, such as CaAl_2_H_2_, ScAl_2_H_2_, and YAl_2_H_2_, can serve as dependable references when experimental data are unavailable. Second, compared to CaAl_2_H_2_ and SrAl_2_H_2_, the positions of Al atoms in ScAl_2_H_2_ and YAl_2_H_2_ show minor variations, whereas the positions of H atoms in ScAl_2_H_2_ and YAl_2_H_2_, especially in YAl_2_H_2_, undergo significant shifts closer to the ab-plane. Consequently, this leads to an increase in the Al-H bond distances in YAl_2_H_2_, potentially weakening the Al-H chemical bond strength. Furthermore, the similar Al-H interatomic distances observed in the three materials, excluding YAl_2_H_2_, suggest that the Al-H bond strength in these three materials is approximately equivalent.

The calculated lattice parameters, unit cell volume, bulk modulus, formation enthalpy, and gravimetric hydrogen storage capacities for the series are presented in Table 2, along with reference values of the lattice parameters for SrAl_2_H_2_ [17,22]. It should be noted that our optimized lattice parameters for SrAl_2_H_2_ show better agreement with the experimental data, with errors of 0.08% and 0.38% for the parameters *a* and *c*, respectively. Meanwhile, the formation enthalpy of the series is calculated using Equation (2):

(2) $$\Delta H=E({\mathrm{XAl}}_{2}H_{2})-E(X)-2E(\mathrm{Al})-E(H_{2})$$

where *E*(XAl_2_H_2_) denotes the total energy per formula unit of XAl_2_H_2_ (X = Ca, Sr, Sc, Y), *E*(X) and *E*(Al) represent the solid-phase elemental energies of X and Al, respectively, and *E*(H_2_) corresponds to the ground-state energy (2.32 Ry) of an isolated H_2_ molecules. The calculated formation enthalpies of −1.46, −1.80, −0.47, and −1.33 eV/f.u. for CaAl_2_H_2_, SrAl_2_H_2_, ScAl_2_H_2_, and YAl_2_H_2_, respectively, demonstrate thermodynamic stability through their negative values. This stability hierarchy, with SrAl_2_H_2_ exhibiting the most negative Δ*H* (−1.80 eV/f.u.), is indicative of enhanced structural robustness and implies that these hydrides may possess feasible synthesis potential under ambient conditions. Moreover, the quantitative evaluation of hydrogen storage efficiency generally relies on gravimetric analysis, a method that quantifies the mass ratio of stored hydrogen relative to the host material’s mass. Gravimetric storage capacity, a key metric for evaluating hydrogen storage performance, represents the mass of hydrogen stored per unit mass of the material and is defined by Equation (3) [34]:

(3) $$C_{\mathrm{wt}\%}=\left(\frac{\left(\frac{H}{M}\right)m_{H}}{m_{\mathrm{Host}}+\left(\frac{H}{M}\right)m_{H}}\times 100\right)\%$$

Here, H/M denotes the ratio of hydrogen atoms to material atoms, while m_Host_ and m_H_ represent the molar mass of the host material and hydrogen, respectively. As shown in Table 2, the gravimetric hydrogen storage capacities of CaAl_2_H_2_, SrAl_2_H_2_, ScAl_2_H_2_, and YAl_2_H_2_ are 1.41 wt%, 0.94 wt%, 1.34 wt%, and 0.93 wt%, respectively. Although the gravimetric hydrogen storage capacity obtained in this research is marginally below the 5.5 wt% target established by the U.S. Department of Energy (DOE), the aluminum-based compounds investigated here offer distinct advantages rooted in aluminum’s abundant natural reserves and cost-effectiveness, which serve as key strengths for practical applications. Furthermore, the hydrogen storage capacity is anticipated to be enhanced through emerging strategies, including doping, catalysis, or nanostructuring [8]. Notably, CaAl_2_H_2_ exhibits a relatively higher gravimetric hydrogen storage capacity due to the lower atomic mass of the Ca atom compared to the other elements. Moreover, desorption temperature can be calculated using the equation presented in [35]:

(4) $$T_{\mathrm{des}}=\frac{\left|\Delta H\right|}{\Delta S}$$

where Δ*H* represents the computed formation enthalpy, and Δ*S* stands for the entropy variation during the dehydrogenation reaction, which is roughly 130.7 J/(mol·K). As shown in Table 2, the desorption temperatures of CaAl_2_H_2_, SrAl_2_H_2_, ScAl_2_H_2_, and YAl_2_H_2_ are 1076 K, 1326 K, 349 K, and 983 K, respectively. The hydrogen desorption temperature of ScAl_2_H_2_ lies well within the acceptable range (233 K–333 K) specified by the DOE [36]. By combining the data from Table 1 and Table 2, it can be observed that for XAl_2_H_2_ materials, there is no direct correlation between the Al-H bond distance and hydrogen desorption temperatures. This observation is consistent with findings reported in previous studies [16,37]. In contrast, the desorption temperatures of the other compounds significantly exceed the upper limit of 333 K, indicating that CaAl_2_H_2_, SrAl_2_H_2_, and YAl_2_H_2_ require higher temperatures for hydrogen release. This may adversely impact the efficiency of hydrogen storage and release processes.

### 3.2. Electronic Properties

The electronic structure of materials is crucial in determining hydrogen adsorption energy, as the quantum-level charge distribution directly influences the binding mechanisms between hydrogen species and storage substrates.

**Figure 3:**
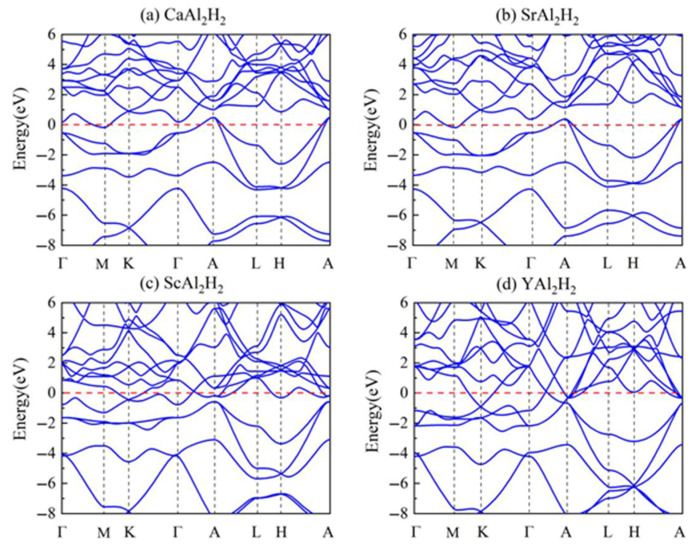
(Color online.) Band structures of (**a**) CaAl_2_H_2_, (**b**) SrAl_2_H_2_, (**c**) ScAl_2_H_2_, and (**d**) YAl_2_H_2_.

The calculated band structures of the XAl_2_H_2_ compounds are presented in Figure 3. The horizontal axis denotes the high-symmetry points in the Brillouin zone, while the vertical axis corresponds to the energy of electron states. Overall, certain bands intersect the Fermi level, which is denoted by the red dashed line. This intersection implies that there is no energy gap for electrons in the valence band to overcome when transitioning to the conduction band. Consequently, all XAl_2_H_2_ compounds exhibit metallic characteristics, a feature that is highly conducive to improving hydrogen storage performance. Specifically, this metallic behavior is expected to significantly enhance hydrogen diffusion kinetics by promoting electron mobility, thereby reducing the activation energy barriers for interstitial hydrogen migration within the crystalline lattice. From Figure 3b, it is evident that the calculated band structure of SrAl_2_H_2_ exhibits remarkable consistency with the results reported in previous studies [22]. Additionally, the band structure distribution of CaAl_2_H_2_ depicted in Figure 3a exhibits a high degree of similarity to that of SrAl_2_H_2_, with the trend characteristics of each energy band along the high-symmetry points being essentially consistent. It can be clearly observed in Figure 3a,b that for both compounds mentioned above, the highest point of the valence band is situated at the A point, where the valence band intersects the Fermi level. Furthermore, the lowest point of the conduction band is positioned at the M point, and the conduction band also crosses the Fermi level in close proximity to this point. However, in contrast to the band structures of the first two compounds, the energy bands of ScAl_2_H_2_ and YAl_2_H_2_ exhibit extensive and complex overlaps near the Fermi level, as illustrated in Figure 3c,d. These overlaps originate from the orbital contributions of the main constituent elements. For instance, Ca in CaAl_2_H_2_, with a valence electron configuration of 4*s*^2^, predominantly contributes electrons from the 4*s* orbital, while Al (3*s*^2^3*p*^1^) and H contribute electrons from their respective 3*s*/3*p* and 1*s* orbitals. This leads to a band structure primarily composed of *s*-like and *p*-like bands. In contrast, Sc in ScAl_2_H_2_, with an electron configuration of 3*d*^1^4*s*^2^, not only contributes electrons from the 4*s* orbital but also introduces significant contributions from the 3*d* orbital, resulting in additional *d*-like bands. Consequently, the band structure of ScAl_2_H_2_ becomes more intricate, with numerous bands crossing the Fermi level. Similarly, since Y in YAl_2_H_2_ has an electron configuration of 4*d*^1^5*s*^2^, its energy bands resemble those of ScAl_2_H_2_.

**Figure 4:**
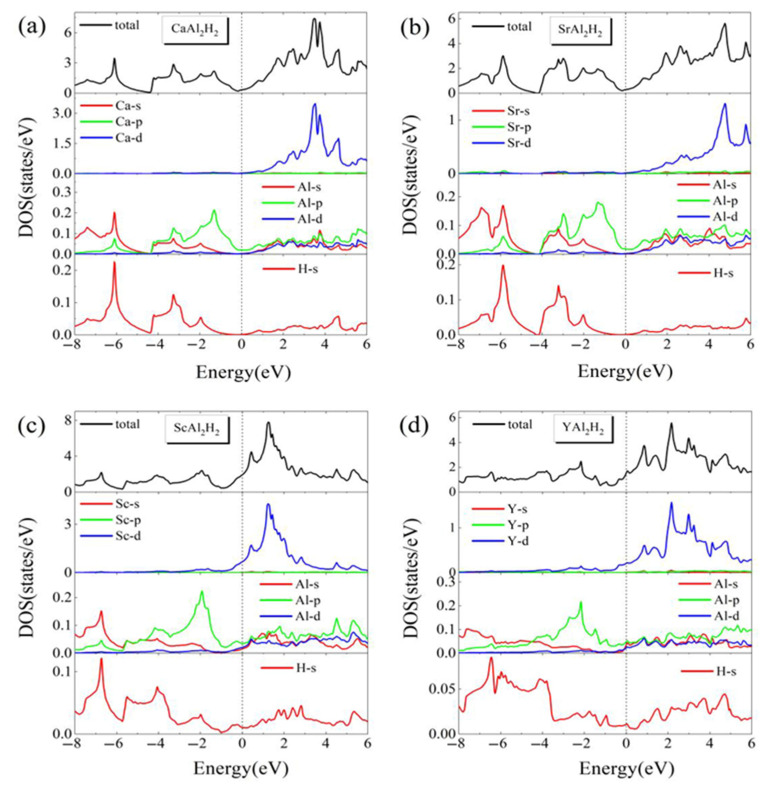
(Color online.) The calculated total and partial DOS of (**a**) CaAl_2_H_2_, (**b**) SrAl_2_H_2_, (**c**) ScAl_2_H_2_, and (**d**) YAl_2_H_2_. The Fermi levels have been set to 0 eV and marked by dot lines.

Figure 4 displays the total densities of states (TDOS) and partial densities of states (PDOS) projected onto the *s*, *p*, and *d* orbitals of X atoms (X = Ca, Sr, Sc, and Y); the *s*, *p*, and *d* orbitals of Al; and the *s* orbitals of H in the series of compounds. As observed in Figure 4a–c, the non-zero DOS at the Fermi level provides conclusive evidence of metallic characteristics for these materials, which is consistent with the band structure analysis results. Firstly, the DOS distribution for SrAl_2_H_2_ closely aligns with those reported in references [20,21,22], thereby validating the reliability of the calculated results. As shown in Figure 4, the X (X = Ca, Sr, Sc, Y) atom predominantly contributes via its *d* orbitals, whereas the Al atom primarily contributes through its *s* and *p* orbitals. Notably, the contribution from the *d* orbitals of Al is relatively minor and predominantly located above the Fermi level. Secondly, as illustrated in Figure 4a,b, the density of states (DOSs) for CaAl_2_H_2_ and SrAl_2_H_2_ exhibits a high degree of similarity due to their belonging to the same group of elements. For CaAl_2_H_2_ and SrAl_2_H_2_, the DOS near the Fermi level is predominantly attributed to the 3*p* orbitals of Al atoms. It is noteworthy that CaAl_2_H_2_ displays an apparent pseudo-band gap nearby the Fermi level, suggesting that, similar to SrAl_2_H_2_, its conductivity is relatively weak. As depicted in Figure 4a, the *d* orbitals of the Ca atom are mainly distributed above the Fermi level, while the *s* and *p* orbitals of the Al atom and the *s* orbital of the H atom are predominantly situated below the Fermi level. In the vicinity of the −8 to −2 eV region, multiple peaks of equivalent energy are observed in the *s* and *p* orbitals of Al and the *s* orbital of H, indicating significant hybridization among these orbitals and the formation of an Al-H covalent bond. Such hybridization also occurs in SrAl_2_H_2_, as shown in Figure 4b. Thirdly, the DOS of ScAl_2_H_2_ shows similarities with those of CaAl_2_H_2_ and SrAl_2_H_2_, particularly in the distribution of atomic states across different energy regions and in the hybridization between Al *s*/*p* orbitals and H *s* orbitals. However, a notable distinction exists in the DOS of ScAl_2_H_2_. In detail, while the Fermi level in CaAl_2_H_2_ and SrAl_2_H_2_ is predominantly influenced by Al 3*p* orbitals, the DOS near the Fermi level in ScAl_2_H_2_ arises from both Al 3*p* and significant Sc 3*d* orbital contributions, as illustrated in Figure 4c. Although other orbitals, such as H *s* and Al *s*/*d*, also contribute, their influence is relatively minor compared to the combined effect of Al 3*p* and Sc 3*d* orbitals. The presence of Sc 3*d* orbitals introduces additional electronic states near the Fermi level, resulting in an increased DOS occupancy and a shift of the pseudo-band gap to lower energies. This leads to enhanced conductivity in ScAl_2_H_2_ compared to CaAl_2_H_2_ and SrAl_2_H_2_. The enhanced DOS occupation at the Fermi level also accounts for the complex band crossings observed near the Fermi level in Figure 3c for ScAl_2_H_2_. In the case of YAl_2_H_2_, as shown in Figure 4d, although the pseudo-band gap becomes less defined, similar changes are observed in the DOS. Specifically, the contribution of Y 4*d* orbitals at the Fermi level increases, and the pseudo-band gap shifts toward lower energy levels, which also indicates a trend of improved conductivity relative to CaAl_2_H_2_ and SrAl_2_H_2_. Additionally, the hybridization between the *s* and *p* orbitals of Al and the *s* orbitals of H in compound YAl_2_H_2_ is significantly reduced. This phenomenon correlates with the increased interatomic distance *d*_Al-H_ observed in structural analysis and the consequent weakening of interactions between Al and H atoms. However, as previously noted, there is no direct correlation between the Al-H bond and the hydrogen desorption temperature in the systems examined nor in certain other multicomponent aluminum hydrides [16,37]. Consequently, although the Al-H hybridization weakens in YAl_2_H_2_, its influence on the hydrogen desorption temperature is not a determining factor.

**Figure 5:**
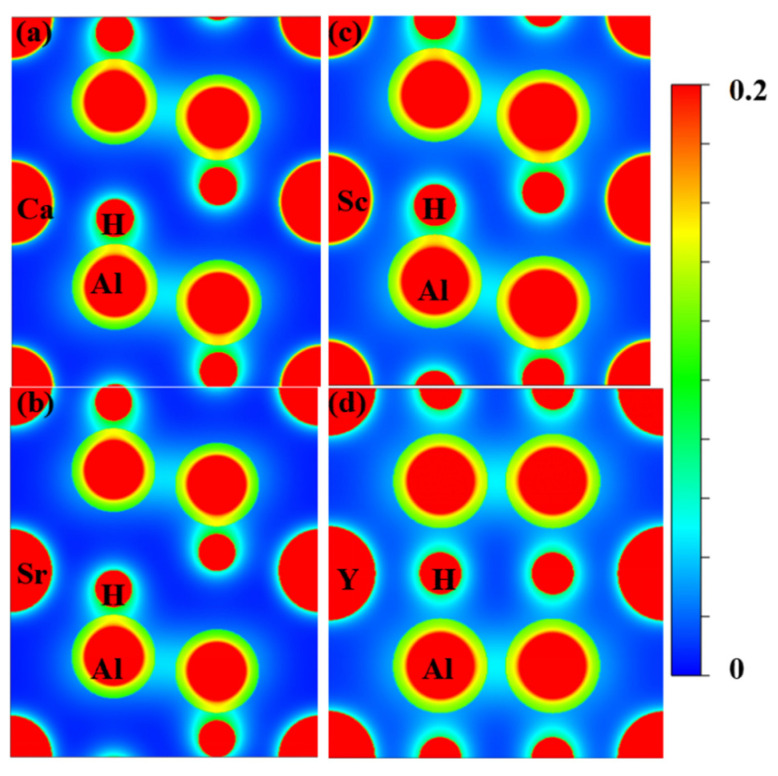
(Color online.) The distribution of charge density of (**a**) CaAl_2_H_2_, (**b**) SrAl_2_H_2_, (**c**) ScAl_2_H_2_, and (**d**) YAl_2_H_2_ in the (1 1 0) plane.

Furthermore, the electron density distributions of XAl_2_H_2_ on the (1 1 0) plane are calculated, as shown in Figure 5. These charge density maps clearly reveal significant charge accumulation between adjacent Al and H atoms, as well as between Al-Al pairs, thereby confirming the presence of covalent bonding interactions. Moreover, our results indicate that all X atoms in each compound exhibit ionic bonding characteristics. Thus, a combination of covalent and ionic bonds in these compounds is revealed. Notably, for YAl_2_H_2_, the charge accumulation between Al and H atoms is substantially reduced compared to CaAl_2_H_2_, SrAl_2_H_2_, and ScAl_2_H_2_, indicating weaker Al-H covalent bonds in YAl_2_H_2_. This observation is consistent with the conclusions drawn from the DOS analysis.

### 3.3. Mechanical Properties

The fundamental mechanical properties of materials play a critical role in both material design and applications. Through computational characterization of the XAl_2_H_2_ hydrides (X = Ca, Sr, Sc, and Y), the six independent single-crystalline elastic constants (*C*_11_, *C*_12_, *C*_13_, *C*_33_, *C*_44_, and *C*_66_) are determined as presented in Table 3. Although no direct experimental or theoretical reference data are currently available, the reliability of the present computational results is validated by incorporating theoretically calculated values for SrAlSiH and CaAlSiH [38]. These compounds possess identical crystallographic structures and are chemically analogous in composition to the studied system, thereby providing a robust basis for comparison. Moreover, it is crucial to note that the stability of the hexagonal phase requires satisfying Born’s mechanical stability conditions [39,40]:

(5) $$\left(C_{11}+2C_{12}\right)C_{33}-2{C_{13}}^{2}>0$$

Upon comparing these values with the stability criteria, it is confirmed that all compounds meet the requirements for mechanical stability assessment. Furthermore, as shown in Table 3, for this series of materials, the slight difference between the values of *C*_11_ and *C*_33_ indicates that the compressive resistance along the *a*-axis and *c*-axis in these compounds is nearly equivalent. However, a notable difference is observed: for YAl_2_H_2_, *C*_11_ is considerably larger than *C*_33_, indicating that the material is more compressible along the *c*-axis direction compared to the *a*-axis. Additionally, the relatively low values of *C*_12_ and *C*_13_ in these compounds imply that when pressure is applied along the a-axis, the materials tend to exhibit shear deformation along the *b*-axis and c-axis of the crystal. Moreover, for the compounds CaAl_2_H_2_, SrAl_2_H_2_, and ScAl_2_H_2_, the elastic constant *C*_44_ is smaller than *C*_66_, indicating that shear deformation is more easily achieved in the (0 0 1) plane compared to the (1 0 0) plane. In contrast, the compound YAl_2_H_2_ shows an opposite trend, with *C*_44_ exceeding *C*_66_, suggesting that shear deformation is more favorably facilitated in the (1 0 0) plane than in the (0 0 1) plane.

In addition, the elastic moduli of the polycrystalline XAl_2_H_2_ (X = Ca, Sr, Sc, and Y) compounds, including bulk modulus (*B*) and shear modulus (*G*), are calculated using the Voigt–Reuss–Hill (VRH) approximations [41]. Young’s modulus (*E*) and Poisson’s ratio (*ν*) are mathematically expressed as follows [39]:

(6) $$E=\frac{9BG}{3B+G}$$

(7) $$\nu =\frac{3B-2G}{2(3B+G)}$$

From Table 4, it can be seen clearly that the computed bulk moduli for CaAl_2_H_2_ (55.35 GPa), SrAl_2_H_2_ (52.44 GPa), ScAl_2_H_2_ (75.47 GPa), and YAl_2_H_2_ (79.15 GPa) exhibit excellent agreement with the values obtained from the B-M equation of state fitting, which are 55.16 GPa for CaAl_2_H_2_, 51.64 GPa for SrAl_2_H_2_, 76.01 GPa for ScAl_2_H_2_, and 74.82 GPa for YAl_2_H_2_, as presented in Table 2. This consistency confirms the reliability of the calculated elastic constants. Our computational results reveal that in the studied hexagonal crystals XAl_2_H_2_ (X = Ca, Sr, Sc, and Y), the relatively smaller lattice parameter ratio *c*/*a* serves as a crucial factor leading to an enhanced bulk modulus. The optimized *c*/*a* ratios follow the order SrAl_2_H_2_ (1.05) > CaAl_2_H_2_ (1.03) > ScAl_2_H_2_ (0.99) > YAl_2_H_2_ (0.95), while the corresponding calculated bulk moduli display an inverse progression: SrAl_2_H_2_ (52.44 GPa) < CaAl_2_H_2_ (55.35 GPa) < ScAl_2_H_2_ (75.47 GPa) < YAl_2_H_2_ (79.15 GPa). The same phenomenon has also been observed in similar systems, such as the compounds XAlSiH (X = Ca, Sr, Ba) reported in Reference [38] and XGaSiH (X = Ca, Sr, Ba) described in Reference [18]. Furthermore, a material possessing both a high shear modulus and Young’s modulus exhibits excellent resistance to deformation and superior stiffness. Among the studied series, it is observed that the shear modulus (*G* = 38.53 GPa) and Young’s modulus (*E* = 98.78 GPa) of ScAl_2_H_2_ are slightly lower than those of the other three compounds, indicating relatively weaker resistance to deformation and reduced stiffness. However, as shown in Table 4, the shear modulus and Young’s modulus values for other aluminum-based hydrides are also presented. A comparative analysis reveals that, although the mechanical moduli of ScAl_2_H_2_ are slightly lower than those of the other three compounds examined in this study, they still exhibit overall superior performance compared to other aluminum-based hydrides such as Rb_2_AlTlH_6_ [42] and NaAlH_3_ [15].

Pugh’s mechanical stability criterion [43] employs the bulk-to-shear modulus ratio (*B*/*G* > 1.75 for ductility) to classify material behavior. Our calculations reveal distinct patterns: CaAl_2_H_2_ (*B*/*G* = 0.99) and SrAl_2_H_2_ (*B*/*G* = 1.25) show intrinsic brittleness, whereas ScAl_2_H_2_ (*B*/*G* = 2.04) and YAl_2_H_2_ (*B*/*G* = 1.79) exhibit superior ductility, as shown in Table 4. Complementary analysis through Poisson’s ratio confirms this contrast in mechanical properties: values below the 0.26 threshold [44] for CaAl_2_H_2_ (*ν* = 0.176) and SrAl_2_H_2_ (*ν* = 0.152) indicate limited plastic deformation capacity, while ScAl_2_H_2_ (*ν* = 0.281) and YAl_2_H_2_ (*ν* = 0.265) surpass this critical value, demonstrating enhanced ductile behavior that aligns consistently with *B*/*G* ratio predictions. Moreover, Poisson’s ratio can serve as a criterion for distinguishing between the characteristics of ionic and covalent bonds in compounds. Specifically, a Poisson’s ratio approaching 1 is indicative of predominantly ionic bonding, whereas a value of approximately 0.25 suggests covalent bonding [45]. The calculated results reveal that covalent bonding dominates in CaAl_2_H_2_ and SrAl_2_H_2_ compounds, while ionic bonding is predominant in ScAl_2_H_2_ and YAl_2_H_2_ compounds. Furthermore, the elastic anisotropic properties of materials are quantitatively assessed using the anisotropy index *A*, which is determined through the following equation [46]:

(8) $$A=\frac{2C_{44}}{C_{11}-C_{12}}$$

**Figure 6:**
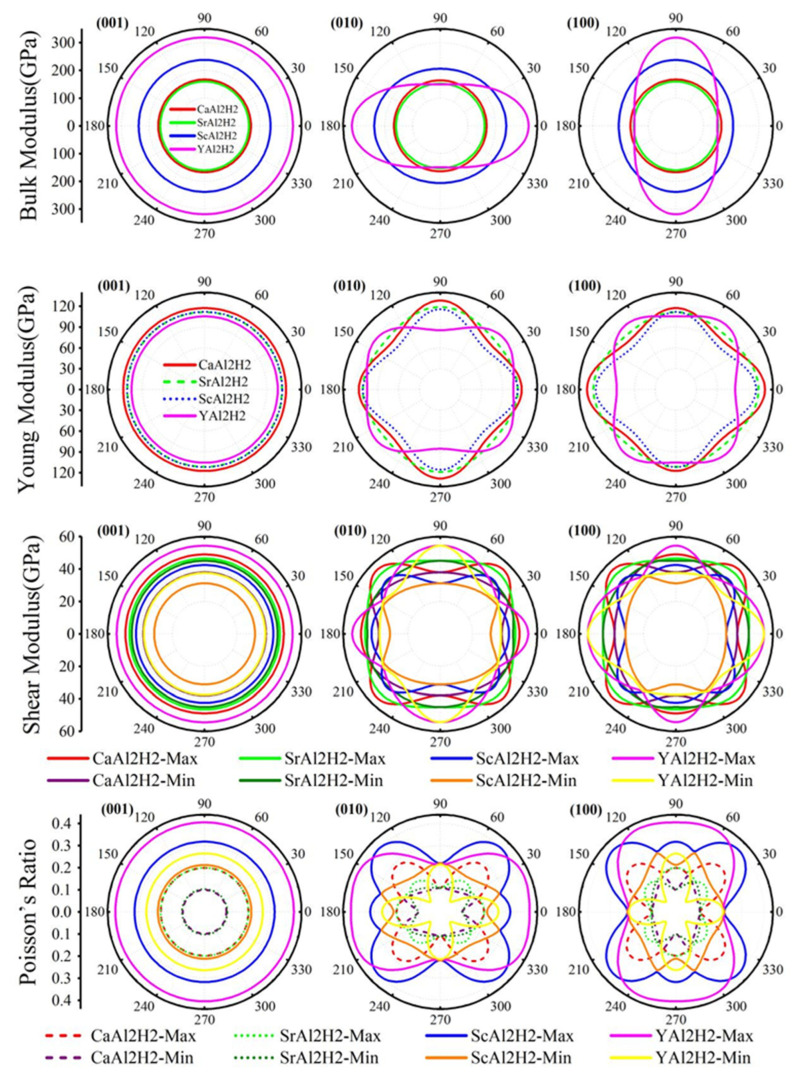
(Color online.) Two-dimensional plots of calculated anisotropy mechanical parameters (bulk, shear, Young’s modulus and Poisson’s ratio) at (0 0 1), (0 1 0) and (1 0 0) plane for XAl_2_H_2_ (X = Ca, Sr, Sc, Y).

*A* value of *A* = 1 indicates perfect isotropy, whereas deviations from unity signify increasing anisotropy. The computed *A* values for CaAl_2_H_2_, SrAl_2_H_2_, ScAl_2_H_2_, and YAl_2_H_2_ are 0.775, 0.152, 0.281, and 0.265, respectively, suggesting pronounced anisotropic characteristics in these materials. To more directly and thoroughly demonstrate the characteristics of elastic anisotropy, Figure 6 presents two-dimensional graphs that show the directional dependence of the bulk modulus, shear modulus, Young’s modulus, and Poisson’s ratio. As shown in Figure 6, apart from the (0 0 1) plane exhibiting relatively isotropic behavior as indicated by its nearly circular plot, the (0 1 0) plane and (1 0 0) plane both display varying degrees of anisotropy, with their plots deviating from circular symmetry to different extents.

In addition, mass density *ρ*, average sound velocity *v*_m_, longitudinal wave sound velocity *v*_l_, transverse wave sound velocity *v*_t_, and Debye temperature *θ*_D_ are shown in Table 3 and can be estimated using the following relationships [39]:

(9) $$\theta_{D}=\frac{h}{k_{B}}{\left[\frac{3n}{4\pi }\left(\frac{N_{A}\rho }{M}\right)\right]}^{\frac{1}{3}}v_{m}$$

(10) $$v_{m}={\left[\frac{1}{3}\left(\frac{2}{v_{t}^{3}}+\frac{1}{v_{l}^{3}}\right)\right]}^{-\frac{1}{3}}$$

(11) $$v_{l}=\sqrt{\left(B+\frac{4}{3}G\right)/\rho }$$

(12) $$v_{t}=\sqrt{G/\rho }$$

Here, *M*, *N*_A_, *ρ*, h, k, and *n* represent the molecular weight, Avogadro’s number, the density of the material, Planck’s constant, Boltzmann’s constant, and the number of atoms in the unit cell, respectively.

The Debye temperature can be regarded as an indicator of the stiffness or rigidity of a solid lattice. The Debye temperature of CaAl_2_H_2_ is calculated to be 620.6 K, significantly higher than that of the other three compounds. This suggests that CaAl_2_H_2_ exhibits superior crystalline lattice rigidity.

### 3.4. Lattice Dynamical and Thermodynamic Properties

**Figure 7:**
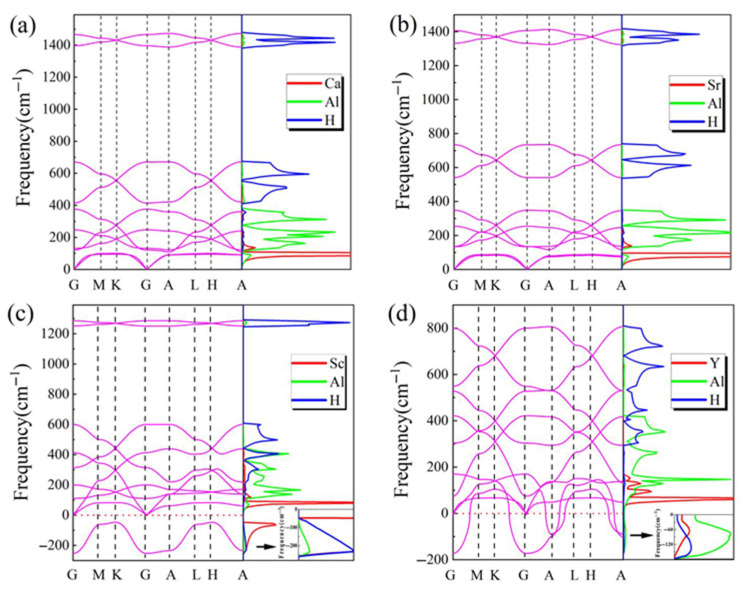
(Color online.) Calculated phonon dispersion and partial phonon density of states of (**a**) CaAl_2_H_2_, (**b**) SrAl_2_H_2_, (**c**) ScAl_2_H_2_, and (**d**) YAl_2_H_2_.

The calculated phonon spectra and projected phonon density of states (PPDOSs) are illustrated in Figure 7a–d, providing valuable insights into the lattice dynamical properties and enabling a deeper exploration of the thermodynamic properties. The absence of imaginary frequencies in the phonon dispersion curves of CaAl_2_H_2_ and SrAl_2_H_2_ confirms their dynamical stability, whereas the presence of imaginary frequencies in those of ScAl_2_H_2_ and YAl_2_H_2_ suggests their potential dynamical instability in the structure. For SrAl_2_H_2_, the phonon dispersion curves are found to be in good agreement with those reported in previous theoretical studies [21,22]. As shown in Figure 7b, the phonon spectrum of SrAl_2_H_2_ is distinctly distributed across three frequency regions: the low-frequency region (0–348.4 cm^−1^), the intermediate-frequency region (540.8–734.5 cm^−1^), and the high-frequency region (1324.4–1412.7 cm^−1^). Most of the phonon DOS in the low-frequency region originates from Al atoms, which predominantly contribute to the range of approximately 100–350 cm^−1^, while the remaining contributions are from Sr atoms that are primarily responsible for the range of about 0–100 cm^−1^. In both the intermediate-frequency and high-frequency regions, the contributions are predominantly from H atoms, with minor contributions from Al atoms. Crystal structures that are similar tend to exhibit phonon dispersion curves with comparable characteristics. As shown in Figure 7a, the phonon dispersion curves for CaAl_2_H_2_ can also be divided into three frequency zones: the low-frequency region (0–376.3 cm^−1^), the intermediate-frequency region (414.2–670.1 cm^−1^), and the high-frequency region (1388.2–1473.2 cm^−1^), respectively. It is noteworthy that for both CaAl_2_H_2_ and SrAl_2_H_2_, in the high-frequency region, the primary contributions predominantly originate from the vibrations between Al and H atoms. This can be primarily attributed to the stronger covalent character of the Al-H bonds. It is observed that in the phonon DOS for both compounds, the contribution of Ca or Sr atoms is only distributed within a narrow frequency range of 0–100 cm^−1^. This indicates that the vibrations of Ca or Sr atoms are more localized compared to those of other atoms. Consequently, this further suggests that the bonding strength of X atoms (e.g., Ca or Sr) is relatively weak, providing only a limited restoring force to sustain interatomic resonance. For ScAl_2_H_2_, as clearly shown in Figure 7c, the phonon dispersion curves exhibit significant imaginary modes throughout the entire Brillouin zone, with the contributions to the phonon DOS associated with these imaginary modes predominantly originating from Sc atoms. For YAl_2_H_2_, as illustrated in Figure 7d, the phonon dispersion curves display substantial imaginary modes across most of the Brillouin zone, except along the high-symmetry directions M-K and L-H. The contributions to the phonon DOS linked to these imaginary modes are primarily attributed to Al atoms, and minor contributions come from Y and H atoms.

Additionally, the hexagonal structure of the *P*-3*m*1 space group belongs to O_3d_ point group. Therefore, as detailed in Table 5, the characters of the irreducible representation for the XAl_2_H_2_ (X = Ca, Sr, Sc, and Y) compounds at the Γ point are determined according to factor group theory [47]. Therein, it is observed that the modes belong to single states characterized by the A representation and double-degenerate states characterized by the E representation. Among the optic modes, these degenerate states are both Raman (R)-active and infrared (IR)-active. The calculated frequencies of modes at the Γ point for CaAl_2_H_2_ and SrAl_2_H_2_ are listed in Table 5. The calculation results for SrAl_2_H_2_ are in good agreement with the available experimental values [48]. However, the data for ScAl_2_H_2_ and YAl_2_H_2_ are excluded because of the presence of significant imaginary modes within the Brillouin zone.

**Figure 8:**
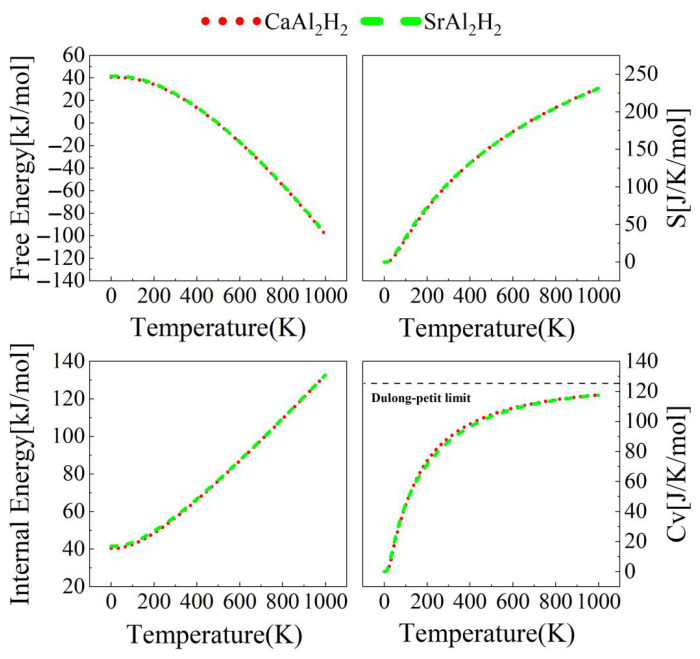
(Color online.) Calculated free energy, internal energy, entropy *S*, and the specific heat at constant volume *C*_v_ as a function of temperature for CaAl_2_H_2_ and SrAl_2_H_2_.

Furthermore, temperature plays a pivotal role in material performance, necessitating an exploration of thermodynamic property variations with temperature. The temperature-dependent behavior of Helmholtz free energy (*F*), internal energy (*E*), entropy (*S*), and constant-volume specific heat (*C*v) is investigated from 0 K to 1000 K using the harmonic approximation, as presented in Figure 8. The computational framework relies on the following equations [39]:

(13) $$F=3nNk_{B}T\int_{0}^{\omega_{\max}}\ln\left(2\sinh\frac{\hbar \omega }{2k_{B}T}\right)g\left(\omega \right)d\omega$$

(14) $$E=3nN\frac{\hbar }{2}\int_{0}^{\omega_{\max}}\omega \coth\left(\frac{\hbar \omega }{2k_{B}T}\right)g\left(\omega \right)d\omega$$

(15) $$S=3nNk_{B}\int_{0}^{\omega_{\max}}\left[\frac{\hbar \omega }{2k_{B}T}\coth\frac{\hbar \omega }{2k_{B}T}-\ln\left\{2\sinh\frac{\hbar \omega }{2k_{B}T}\right\}\right]g\left(\omega \right)d\omega$$

(16) $$C_{V}=3nNk_{B}\int_{0}^{\omega_{\max}}{\left(\frac{\hbar \omega }{2k_{B}T}\right)}^{2}\csc h^{2}\left(\frac{\hbar \omega }{2k_{B}T}\right)g\left(\omega \right)d\omega$$

Here, *k*_B_ is the Boltzmann constant, *ħ* is the reduced Planck constant, *n* is the number of atoms per unit cell, *N* is the number of unit cell, *T* is the temperature, *ω* is the phonon frequency, and *ω*_max_ is the maximum phonon frequency, respectively. In addition, the normalized phonon DOS *g*(*ω*) satisfies the normalization condition [39]:

(17) $$\int_{0}^{\omega_{\max}}g\left(\omega \right)d\omega =1$$

The thermodynamic data of ScAl_2_H_2_ and YAl_2_H_2_ are omitted in Figure 8, as the formulas used to calculate these data rely on the phonon DOS, and the phonon spectra of these two compounds exhibit imaginary frequencies. It is worth noting that the thermodynamic data for CaAl_2_H_2_ and SrAl_2_H_2_ are very close, with their curves nearly overlapping in Figure 8, attributable to their similar chemical compositions. As shown in Figure 8, the computed free energy *F* is found to decrease steadily as temperature rises. Meanwhile, the internal energy *E* increases linearly at temperatures above room temperature. At absolute zero, the internal energy *E* equals the free energy *F*, a value referred to as the zero-point energy *E*_0_, which can be calculated via the equation below [39]:

(18) $$F_{0}=E_{0}=3nN\int_{0}^{\omega_{\max}}\frac{\hbar \omega }{2}g\left(\omega \right)d\omega$$

The zero-point energies *E*_0_ for CaAl_2_H_2_ and SrAl_2_H_2_ are calculated as 40.49 kJ/mol and 41.51 kJ/mol, respectively. Notably, the entropy *S* values exhibit a monotonic increase with temperature. At 300 K, the entropies of CaAl_2_H_2_ and SrAl_2_H_2_ are 104.4 J/(mol∙K) and 105.1 J/(mol∙K), respectively. For the constant-volume heat capacity *C*_v_, both the compounds show a temperature-dependent increase, following Debye’s *T*^3^ law in the low-temperature regime. At 300 K, the *C*_v_ values for CaAl_2_H_2_ and SrAl_2_H_2_ are determined to be 89.0 J/(mol∙K) and 86.4 J/(mol∙K), respectively. As temperature continues to rise, *C*_v_ gradually approaches the classical limit predicted by the Dulong–Petit law: *C*_v_ = 3*nR* = 3 × 5 × 8.31 = 124.65 J/(mol∙K).

## 4. Conclusions

In summary, a comprehensive investigation was carried out on the structural, hydrogen storage, electronic, mechanical, lattice dynamics, and thermodynamic properties of the Zintl phase hydrides XAl_2_H_2_ (X = Ca, Sr, Sc, and Y) using the full-potential linearized augmented plane wave (FP-LAPW) method within the framework of density functional theory (DFT). The optimized structural parameters (*a*, *c*, and *c*/*a*) for SrAl_2_H_2_ are in good agreement with the experimental data and other theoretical results, thereby validating the reliability of the structural calculations for CaAl_2_H_2_, ScAl_2_H_2_, and YAl_2_H_2_ reliability. The calculated formation enthalpies of −1.46, −1.80, −0.47, and −1.33 eV/f.u. for CaAl_2_H_2_, SrAl_2_H_2_, ScAl_2_H_2_, and YAl_2_H_2_, respectively, indicate their thermodynamic stability due to the negative values. The gravimetric hydrogen storage capacity of CaAl_2_H_2_ is 1.41%, which is the highest among the studied systems. In comparison, the capacities of SrAl_2_H_2_, ScAl_2_H_2_, and YAl_2_H_2_ are 0.94%, 1.34%, and 0.93%, respectively. The hydrogen desorption temperatures of CaAl_2_H_2_, SrAl_2_H_2_, ScAl_2_H_2_, and YAl_2_H_2_ are 1076 K, 1326 K, 349 K, and 983 K, respectively. Electronic structure analysis reveals that all these compounds exhibit metallic characteristics and feature a combination of covalent and ionic bonding. CaAl_2_H_2_ and SrAl_2_H_2_ exhibit an apparent pseudo-band gap near the Fermi level, suggesting relatively weak conductivity compared to ScAl_2_H_2_ and YAl_2_H_2_. The mechanical stability of the materials is confirmed by satisfying Born’s criteria. ScAl_2_H_2_ and YAl_2_H_2_ exhibit good ductility, whereas CaAl_2_H_2_ and SrAl_2_H_2_ display intrinsic brittleness. However, all these materials show anisotropic characteristics to varying degrees. The absence of imaginary frequencies in the phonon dispersion curves of CaAl_2_H_2_ and SrAl_2_H_2_ confirms their dynamical stability, whereas the presence of imaginary frequencies in the phonon dispersion curves of ScAl_2_H_2_ and YAl_2_H_2_ indicates potential dynamical instability in these structures. Furthermore, for CaAl_2_H_2_ and SrAl_2_H_2_, we calculated the phonon frequencies at the center of the first Brillouin zone, identifying Raman-active and infrared-active vibrational modes. In addition, we provide temperature-dependent thermodynamic properties, including Helmholtz free energy, internal energy, entropy, and heat capacity. Overall, our investigation into hexagonal XAl_2_H_2_ compounds reveals that CaAl_2_H_2_ possesses promising properties as a hydrogen storage material. This study investigated the fundamental physical properties of the XAl_2_H_2_ system for solid-state hydrogen storage, providing a theoretical foundation for understanding the material’s characteristics and facilitating further research in this field.

## Figures and Tables

**Table 1:** The internal coordinates of Al and H atoms and the shortest interatomic distances of Al-H for XAl_2_H_2_ (X = Ca, Sr, Sc, Y).

Compounds	Al	H	*d*_Al-H_ (Å)	Refs.
CaAl_2_H_2_	(1/3, 2/3, 0.456027)	(1/3, 2/3, 0.085312)	1.697	Present
SrAl_2_H_2_	(1/3, 2/3, 0.460439)	(1/3, 2/3, 0.097497)	1.716	Present
SrAl_2_H_2_	(1/3, 2/3, 0.4589)	(1/3, 2/3, 0.0976)	1.71	Exp. [17]
SrAl_2_H_2_	(1/3, 2/3, 0.4608)	(1/3, 2/3, 0.0964)	1.721	Theo. [22]
ScAl_2_H_2_	(1/3, 2/3, 0.441807)	(1/3, 2/3, 0.033534)	1.720	Present
YAl_2_H_2_	(1/3, 2/3, 0.500094)	(1/3, 2/3, 0.000166)	2.067	Present

**Table 2:** The lattice constants (*a* and *c*), *c*/*a* ratio, unit cell volume (*V*_0_), bulk modulus (*B*_0_) and its pressure derivative *B*’, formation enthalpy (Δ*H*), gravimetric hydrogen storage capacities (C_wt_%), and desorption temperature *T*_des_ of XAl_2_H_2_ (X = Ca, Sr, Sc, Y).

Compounds	*a*	*c*	*c/a*	*V*_0_	*B*_0_	*B’*	Δ*H*	C_wt_%	*T*_des_	Refs.
	(Å)	(Å)		(Å^3^)	(GPa)		(eV/f.u.)		(K)	
CaAl_2_H_2_	4.4328	4.5773	1.03	77.90	55.16	3.80	−1.46	1.41	1076	Present
SrAl_2_H_2_	4.5323	4.7395	1.05	84.18	51.64	3.93	−1.80	0.94	1326	Present
SrAl_2_H_2_	4.5283	4.7215	1.04							Exp. [17]
SrAl_2_H_2_	4.528	4.722	1.04							Theo. [22]
ScAl_2_H_2_	4.2343	4.2123	0.99	65.41	76.01	3.94	−0.47	1.34	349	Present
YAl_2_H_2_	4.3440	4.1364	0.95	67.60	74.82	4.01	−1.33	0.93	983	Present

**Table 3:** Calculated elastic constants (GPa) of XAl_2_H_2_ (X = Ca, Sr, Sc, Y).

Compounds	*C*_11_	*C*_12_	*C*_13_	*C*_33_	*C*_44_	*C*_66_	Refs.
CaAl_2_H_2_	124.21	26.30	16.31	131.92	37.94	48.96	Present
SrAl_2_H_2_	118.20	25.15	15.71	122.46	45.09	46.52	Present
ScAl_2_H_2_	137.37	52.43	41.60	133.95	31.16	42.47	Present
YAl_2_H_2_	146.95	71.84	46.83	105.56	54.40	37.54	Present
CaAlSiH	175.6237	39.1212	23.7807	94.061	43.1177	68.25125	Theo. [38]
SrAlSiH	163.2624	32.5895	27.2531	97.4601	46.2550	65.3364	Theo. [38]

**Table 4:** Calculated bulk modulus (*B*), shear modulus (*G*), Young’s modulus (*E*), Pugh ratio (*B*/*G*), Frantsevich ratio (*G*/*B*), Poisson’s ratio (*ν*), anisotropy factor (*A*), density *ρ* (g/cm^3^), Debye temperature (*θ*_D_), transverse elastic wave velocity (*v*_t_), longitudinal elastic wave velocity (*v*_l_), and average wave velocity (*v*_m_) of XAl_2_H_2_ (X = Ca, Sr, Sc, Y).

	**Present Work**				**Other Al-Base Hydrides**			
**Compounds**	**CaAl_2_H_2_**	**SrAl_2_H_2_**	**ScAl_2_H_2_**	**YAl_2_H_2_**	**CaAlSiH [38]**	**SrAlSiH [38]**	**Rb_2_AlTlH_6_ [42]**	**NaAlH_3_ [15]**
*B* (GPa)	55.35	52.44	75.47	79.15	65.350	64.468	27.4	43.669
*G* (GPa)	45.77	47.38	38.53	43.97	53.091	52.919	18.2	8.702
*E* (GPa)	107.64	109.23	98.78	111.30	125.332	124.650	44.6	24.479
*B*/*G*	1.21	1.11	1.96	1.80	1.2309	1.2182	1.50	5.018
*G*/*B*	0.83	0.90	0.51	0.56				
*ν*	0.176	0.152	0.281	0.265	0.180	0.177	0.23	0.407
*A*	0.775	0.969	0.734	1.450			0.72	
*ρ*	2.05	2.83	2.56	3.56				
*θ*_D_ (K)	620.6	521.9	546.0	488.6	606.615	502.165	208.18	
*v*_t_ (m/s)	4727.66	4089.59	3877.55	3514.88	4756.12	4048.46	1870.3	
*v*_l_ (m/s)	7538.60	6388.36	7035.45	6221.7	7616.09	6466.87	3160.11	
*v*_m_ (m/s)	5206.06	4492.98	4321.33	3909.41	5239.81	4458.98	2071.09	

**Table 5:** Vibrational assignments and phonon frequencies *ω* (cm^−1^) at zone-center (Γ point) for CaAl_2_H_2_ and SrAl_2_H_2_. The notations IR and R refer to infrared-active and Raman-active modes, respectively.

**The Point Group: O_3d_ (−3m)**			
$\Gamma_{\mathrm{acoustic}}=A_{2u}⊕E_{u}$ $\Gamma_{\mathrm{optic}}=2A_{1g}⊕2A_{2u}⊕2E_{u}⊕2E_{g}$			
**Mode**	**CaAl_2_H_2_**	**SrAl_2_H_2_**	**SrAl_2_H_2_ [48]**
A_2u_ (IR)	132, 1397	134, 1332	132, 1333
E_u_ (IR)	121, 414	135, 541	143, 593
A_1g_ (R)	247, 1466	254, 1406	267, 1412
E_g_ (R)	376, 670	348, 735	377, 765

## Data Availability

The original contributions presented in this study are included in the article. Further inquiries can be directed to the corresponding author.
